# Seasonal Shift in Climatic Limiting Factors on Tree Transpiration: Evidence from Sap Flow Observations at Alpine Treelines in Southeast Tibet

**DOI:** 10.3389/fpls.2016.01018

**Published:** 2016-07-13

**Authors:** Xinsheng Liu, Yuqin Nie, Tianxiang Luo, Jiehui Yu, Wei Shen, Lin Zhang

**Affiliations:** ^1^College of Tourism and Territorial Resources, Jiujiang University, JiujiangJiangxi Province, China; ^2^Key Laboratory of Alpine Ecology and Biodiversity, Institute of Tibetan Plateau Research, Chinese Academy of SciencesBeijing, China; ^3^CAS Center for Excellence in Tibetan Plateau Earth SciencesBeijing, China

**Keywords:** alpine treeline, transpiration, sap flow, soil temperature, southeast Tibet

## Abstract

Alpine and northern treelines are primarily controlled by low temperatures. However, little is known about the impact of low soil temperature on tree transpiration at treelines. We aim to test the hypothesis that in cold-limited forests, the main limiting factors for tree transpiration switch from low soil temperature before summer solstice to atmospheric evaporative demand after summer solstice, which generally results in low transpiration in the early growing season. Sap flow, meteorological factors and predawn needle water potential were continuously monitored throughout one growing season across Smith fir (*Abies georgei* var. *smithii*) and juniper (*Juniperus saltuaria*) treelines in southeast Tibet. Sap flow started in early May and corresponded to a threshold mean air-temperature of 0°C. Across tree species, transpiration was mainly limited by low soil temperature prior to the summer solstice but by vapor pressure deficit and solar radiation post-summer solstice, which was further confirmed on a daily scale. As a result, tree transpiration for both tree species was significantly reduced in the pre-summer solstice period as compared to post-summer solstice, resulting in a lower predawn needle water potential for Smith fir trees in the early growing season. Our data supported the hypothesis, suggesting that tree transpiration mainly responds to soil temperature variations in the early growing season. The results are important for understanding the hydrological response of cold-limited forest ecosystems to climate change.

## Introduction

Alpine and northern treelines, which are a conspicuous vegetation boundary between forest and tundra ecosystems, are widely thought to be controlled by temperature (Tranquillini, 1979; Körner, 2003). They have received particular attention in the context of climate change since observations and projections consistently suggest that mountainous and boreal regions are undergoing a strong climate warming (Ipcc, 2014). Therefore, knowledge of temperature-mediated tree physiology processes in cold-limited forests, such as tree growth (Rossi et al., 2008; Gruber et al., 2009), root growth (Alvarez-Uria and Körner, 2007), and carbon gain (Wieser et al., 2010; Kong et al., 2012) are essential in forecasting the treeline responses to future warming. In particular, climate warming is also expected to change hydrological functioning of cold-limited forest ecosystems by altering transpiration (Saxe et al., 2001; Barnett et al., 2005; Wieser et al., 2015). Nevertheless, to date, transpiration patterns of treeline forests and its environmental controls remain poorly understood and detailed investigations are urgently needed (Wieser and Tausz, 2007; Brito et al., 2014, 2015).

In recent decades, sap flow techniques allow us to monitor tree transpiration of remote sub-alpine (e.g., Hu et al., 2010; Leuzinger and Bader, 2012) and treeline forests (e.g., Anfodillo et al., 1998; Brito et al., 2014, 2015; Wieser et al., 2014, 2015), enhancing our understanding of seasonal tree water use and its association with meteorological factors. It is expected that tree transpiration of cold-limited forests will be increased under a warmer climate. Many experiments have examined the response of tree transpiration to soil warming, but contradictory conclusions including no effect on black spruce [*Picea mariana* (Mill.) BSP] in northern Canada (Van Herk et al., 2011) vs. positive impact on *Pinus cembra* in an Alpine treeline (Wieser et al., 2015) and Scots pine (*Pinus sylvestris* L.; Mellander et al., 2004) and Norway spruce [*Picea abies* (L.) Karst.; Bergh and Linder, 1999] in northern Sweden were found. Moreover, such contradictory results were even apparent among experimental years with varying timing of soil warming (May vs. July) within a site (Wieser et al., 2015). These discrepancies clearly imply that the controls on tree transpiration may vary in different growing phases. However, to our knowledge, no attention has been devoted to the seasonal variability in the environment dependency of tree transpiration in treeline forests across different growing phases. Such knowledge is crucial for predicting future hydrological functioning in cold-limited forest ecosystems.

In cold-limited forests, evidence has shown that seasonal photosynthetic capacity (Bauerle et al., 2012), annual canopy CO_2_ uptake (Monson et al., 2002) and stem growth (Rossi et al., 2006; Duchesne et al., 2012; Zhai et al., 2012) synchronously peaked around summer solstice, indicating that the transition period from spring to early summer (i.e., the early growing season before summer solstice) is important in regulating tree physiology processes of woody plants in cold environments. However, little is known about the process and influential factors involved in tree transpiration during this period. Due to the presence of ground snow cover, delayed soil thawing is common during the early growing season, which will be intensified by reduced snow cover under a warmer winter (Decker et al., 2003; Mellander et al., 2005). Meanwhile, an extraordinary overheating of tree leaves above ambient air temperature caused by excessive radiation amplified by reflection from snow, leads to atmospheric evaporative demands that are comparable to summer (Wieser and Tausz, 2007). Hence, during the early growing season, trees are exposed to strong atmospheric evaporative demand together with low soil temperature. The inhibitory effects of low soil temperature on transpiration have been demonstrated and modeled for various boreal forests. For instance, Repo et al. (2008) showed that frozen soils effectively blocked water uptake and consequent trunk sap flow of Scots pine saplings using laboratory experiments. Mellander et al. (2006) suggested that low soil temperature during the spring–early summer heavily restricted transpiration of Scots pine in Sweden using the coupled heat and mass transfer model for the soil-plant-atmosphere system (COUP) model. However, *in situ* measurements of sap flow of treeline forest trees are seldom conducted during the transition period from spring to early summer, rendering the controls on tree transpiration unknown.

Southeast Tibet, which is characterized by a cold and moist climate, has the world’s highest elevation treeline (Sheng et al., 2014). In the Sergyemla Mountains, Smith fir (*Abies georgei* var. *smithii*) and juniper (*Juniperus saltuaria*) oppositely dominate on slopes of a valley where annual mean air-temperature and soil warming date differ greatly and annual precipitation is similar (Liu and Luo, 2011). According to local instrumental climatic data, a significant warming trend since the 1960s was observed, which has been recorded in tree-ring width chronologies of Smith fir trees (Liang et al., 2009) and *Rhododendron* shrubs (Liang and Eckstein, 2009; Kong et al., 2012). This provides an ideal natural laboratory for examining the intra-annual variations in transpiration and its association with environmental factors in order to give new insights on temperature affected hydrological functioning in cold-limited forests. Such knowledge and its difference between tree species may also be useful for understanding the formation of the unique treeline distribution pattern in southeast Tibet. In this study, we hypothesize that in cold-limited forests, the main limiting factors for tree transpiration switch from low soil temperature before summer solstice to atmospheric evaporative demand [i.e., vapor pressure deficit (*D*) and solar radiation (*R*)] after summer solstice, which generally results in low transpiration in the early growing season. To test the hypothesis, 1-year sap flow density and relevant meteorology data were observed across Smith fir and juniper treelines in southeast Tibet, and predawn needle water potential (Ψ_pd_) were measured for Smith fir trees over one growing season. Our aims are to: (1) identify the seasonal patterns of sap flow density and Ψ_pd_ across tree species; (2) examine if the sensitivity of diurnal sap flow density to *D* differs among growing-season months; and (3) investigate the temporal variability in the environmental dependency of tree transpiration across pre- and post-summer solstice growing phases.

## Materials and Methods

### Study Sites

This study was conducted on opposite slopes (north-facing vs. south-facing) of a U-shaped valley at the peak of the Sergyemla Mountains (29°36′N, 94°36′E) in southeast Tibet. *A. georgei* var. *smithii* and *J. saltuaria* were the dominant tree species of treelines on the north- and south-facing slopes, respectively. Along both slopes, vegetation changes from sub-alpine and treeline forests (tree height >4 m and canopy coverage >40%) to alpine shrublands and grasslands.

In 2005, two long-term plots (50 m × 50 m) were established at both treeline forests. The stand basal area and mean tree height were 39.7 m^2^ ha^-1^ and 10.2 ±1.0 m for Smith fir and 39.8 m^2^ ha^-1^ and 7.6 ± 0.5 m for juniper. Four automatic weather stations were installed at the sites for treeline forests of Smith fir (4320 m) and juniper (4425 m) and above both treelines. According to the meteorological data during 2005–2012, similar annual precipitation (850–940 mm) and growing-season mean air-temperature (6.5–6.6°C) were found at both treelines, but annual mean air-temperature differed by 2.0°C between non-forested sites with a warmer climate on the south-facing slope. The spring soil warming dates differed by up to 20–30 days between north- and south-facing slopes, while the daily mean soil moisture across both slopes was typically >20% during the growing season. Detailed information on climatic data is found in Liu and Luo (2011) and Kong et al. (2012).

### Environmental Measurements

Meteorological factors at Smith fir and juniper treelines were continuously monitored with a variety of sensors since August 2005. A combined probe with vented radiation shield at a height of 3 m above the ground was used to measured air temperature and relative humidity (MP101A, Rotronic Inc., USA). Soil temperature (S-TMB-M002, with accuracy ± 0.2°C) and volumetric soil moisture content (S-SMA-M003, with accuracy ± 3%) were measured at soil depths of 5, 20, and 60 cm. Additionally, solar radiation (CM3, Kipp & Zonen, Netherlands), precipitation (7852M-AB, Wittich & Visser, Netherlands), and snow depth (260-700, NovaLynx Corp., USA) were observed at a low shrubland near the Smith fir treeline and at a grassland near the juniper treeline. Data were recorded every 15 min and hourly averages were recorded in a HL20 data logger (Jauntering Inc., Taiwan). *D* for both treelines was calculated from measured air temperature and relative humidity (Sheng et al., 2003).

### Sap Flow Measurements

At each treeline site, 12 mature and healthy trees were selected for sap flow density measurement (**Table 1**). Smith fir and juniper trees had an average height of 8.6 ± 1.4 and 7.3 ± 0.6 m, diameter at breast height (DBH) of 25.2 ± 8.4 and 21.8 ± 8.4 cm, and stem age of 130 ± 37 and 208 ± 62 years, respectively. Sap flow densities were monitored by the heat dissipation approach according to Granier (1987). Each sensor consisted of a pair of probes that were 20–60 mm in length and 2 mm in diameter, and a copper-constantan thermocouple was placed in each probe (SF-L, Ecomatik, Germany). Given the potential underestimation of sap flow density when the probe length is longer than the sapwood depth, an appropriate length of probe was used according to the sapwood depth of each monitored tree (Clearwater et al., 1999). Two pieces of bark (ca. 15 mm × 15 mm) on the north-facing side were peeled off, and the pair of probes was inserted into the sapwood about 15 cm vertically apart from each other at breast height (Wieser et al., 2014). The upper probe was continuously heated by a heating wire that was supplied with a 0.2–0.6 W (0.1 W for 10-mm long sensors) direct current (12 V, 65 Ah), whereas the lower probe was unheated providing the reference temperature. The temperature differences between the two probes were measured in 30 s intervals, and 30 min averages were stored on a data logger (CR1000, Campbell Scientific Inc., Logan, UT, USA) with a multiplexer (AM16/32A, Campbell Scientific Inc.). In addition, each sensor was wrapped with waterproof silicone and covered with a radiation shield (Styrofoam sheets with aluminum foil) to avoid thermal influences from solar radiation and physical damage from branches falling. Sap flow density was continuously monitored throughout the growing season in 2012 (May to October). Unfortunately, the sap flow density observation data were lost for five Smith fir trees because of technical problems and power failure (**Table 1**).

**Table 1:** Diameter at breast height (DBH), tree height, tree age, and maximum daily sap flow (Max. *F*_d_) observed during the monitoring period of 2012.

Tree no.	DBH (cm)	Height (m)	Age (year)	Max. *F*_d_ (g cm^-2^ d^-1^)
**Smith fir**
S1	23.6	9.0	122	30.5
S2	40.0	10.5	194	15.7
S3	24.1	9.0	118	39.9
S4	21.7	7.6	107	58.8
S5	32.6	10.0	167	55.6
S6	19.4	7.3	109	14.7
S7	15.1	6.6	90	19.0
**Juniper**
J1	23.9	7.4	228	9.8
J2	12.2	7.6	130	25.6
J3	22.9	7.8	228	51.5
J4	22.8	8.2	231	15.5
J5	23.7	8.1	230	8.8
J6	19.4	7.0	192	15.6
J7	45.2	7.0	369	12.1
J8	17.8	7.1	179	29.7
J9	20.1	6.5	195	17.3
J10	17.4	6.7	175	29.1
J11	13.1	6.4	132	24.6
J12	22.8	7.9	210	4.8

According to the empirical relationship between sap flow density and observed temperature difference between probes established by Granier (1987) and further revalidated by Clearwater et al. (1999), sap flow densities were calculated as:

$$F_{\text{d}}\;=\;119\;\times \;{\left[(\Delta T_{\text{m}}\;-\;\Delta T)/\Delta T\right]}^{1.231}$$

where *F*_d_ is sap flow density (g m^-2^ s^-1^), Δ*T* is the measured temperature difference between the two probes, and Δ*T*_m_ is the maximum temperature difference when *F*_d_ is near 0 (i.e., no-transpiration state). In this study, we assumed that tree transpiration is 0 at night. This is because night-time vapor pressure deficits were low at high elevations, and night-time courses of temperature between probes were balanced which suggests that the recharge of stem water storage has finished. Therefore, Δ*T*_m_ was determined at every night and served as a reference for the next day (Wieser et al., 2014, 2015).

### Needle Water Potential

To understand the seasonal variation in leaf water relations, we measured Ψ_pd_ at the treeline and sub-alpine Smith fir forest sites during the growing season. Ψ_pd_ was observed between 3:00 and 7:00 am on bright days. At each site, six Smith fir trees were selected (DBH and height ranged from 11–61 cm and 7–15 m, respectively), the 1-year-old needles of sun-exposed branches were collected from upper and lower canopies by pole tree pruners. Needle samples were immediately sealed in the psychrometer chamber (C-52 Sample Chamber, Wescor Inc., Logan, UT, USA) which was connected to a portable PSYPRO water potential system (Wescor Inc., Logan, UT, USA). After samples were equilibrated for 10 min, the readings were recorded by the PSYPRO water potential system. In this study, we did not measure the Ψ_pd_ of juniper trees due to the difficulty in sampling the 1–2 mm scale leaves, which may have affected the accuracy of the Ψ_pd_ measurements.

### Data Analysis

In this study, sap flow density and environmental data were collected in 30 and 60 min intervals, respectively. Thus, sap flow density (*F*_d_, g cm^-2^ d^-1^) was summed within a day to harmonize the two data sets and minimize the potential effect of stem internal water storage on the relationships between transpiration and environmental conditions (Oren et al., 1998; Ewers et al., 1999; Wieser et al., 2014, 2015).

Generally, a large variation in sap flow density exists among trees of different size. In this study, we focused on the comparison of transpiration regulators between different growing phases rather than the absolute amount of tree water use. Hence, normalized *F*_d_ was used to minimize the differences among monitored trees. Normalized *F*_d_ was determined by dividing all *F*_d_ for each monitored tree by the tree-specific maximum *F*_d_ during the entire observation period (**Table 1**). Consequently, each tree had a maximum normalized *F*_d_ of 1 and averages calculated from replicates within species were reasonable, which permitted a better comparison of *F*_d_ to environmental conditions (Luis et al., 2005; Du et al., 2011; Brito et al., 2014; Wieser et al., 2014).

To investigate whether the controls on tree transpiration differ before and after summer solstice, moving correlation analysis was applied to evaluate the temporal variability of the relationships between tree transpiration and environmental variables [mean soil temperature at 5 cm (ST_5_), mean air temperature (AT_mean_), *D*, and *R*] throughout the entire growing season. Pearson correlation coefficients were successively computed with a 10-day window, which provided a sufficiently fine resolution. Second, the entire growing season was subdivided into the pre- and post-summer solstice growing phases, and the limiting factors to tree transpiration were separately analyzed. The correlations between normalized *F*_d_ and environmental variables of ST_5_, AT_mean_, *D*, and *R* were fitted with a simple linear model (*y* = *a* + *bx*), which was used for examining how the sensitivity of normalized *F*_d_ to environmental variables differs between pre- and post-summer solstice growing phases across tree species (consistent responses or variable responses). Further, partial correlation analysis of multiple linear regression was used for assessing relative influences of temperature (AT_mean_, ST_5_) and atmospheric evaporative demand (*D, R*) variables on normalized *F*_d_ across tree species and growing phases. Because AT_mean_ and ST_5_ (*r* = 0.69–0.98, *P* < 0.001), and *D* and *R* (*r* = 0.84–0.90, *P* < 0.001) were both strongly correlated, each of the temperature and atmospheric evaporative demand variables were grouped in the partial correlation analysis. In the above analysis, data in rainy days (daily rainfall >5 mm) were excluded because transpiration was greatly reduced or stopped on rainy days (Granier, 1987; Zhang et al., 1999). For each tree species, we used independent sample *t*-tests to test the differences in mean normalized *F*_d_ between pre- and post-summer solstice growing phases.

To understand the influence of soil temperature on transpiration on a daily scale, we selected a bright day in each month from May to August with different soil temperature conditions. Differences in slopes of the relationship between diurnal sap flow density and *D* among different growing-season months (sap flow density as a dependent variable, *D* as a covariate, and date as a grouping variable) were tested using analysis of covariance (ANCOVA) in a general linear model framework. ANCOVA was applied to test whether there were differences in slopes and intercepts (slope elevations) between regression lines.

To examine the relationships between Ψ_pd_ and ST_5_, the data were fitted with a simple linear model (*y* = *a* + *bx*). Differences in Ψ_pd_ of Smith fir trees among observational dates were assessed by one-way analysis of variance and the Tukey comparison (the Tukey–Kramer test is performed by SPSS software when group sizes are unequal).

All statistical analysis were performed using the SPSS 16.0 for Windows (SPSS Inc., Chicago, USA), and all significant differences were at *P* < 0.05.

## Results

### Environment Factors

**FIGURE 1:**
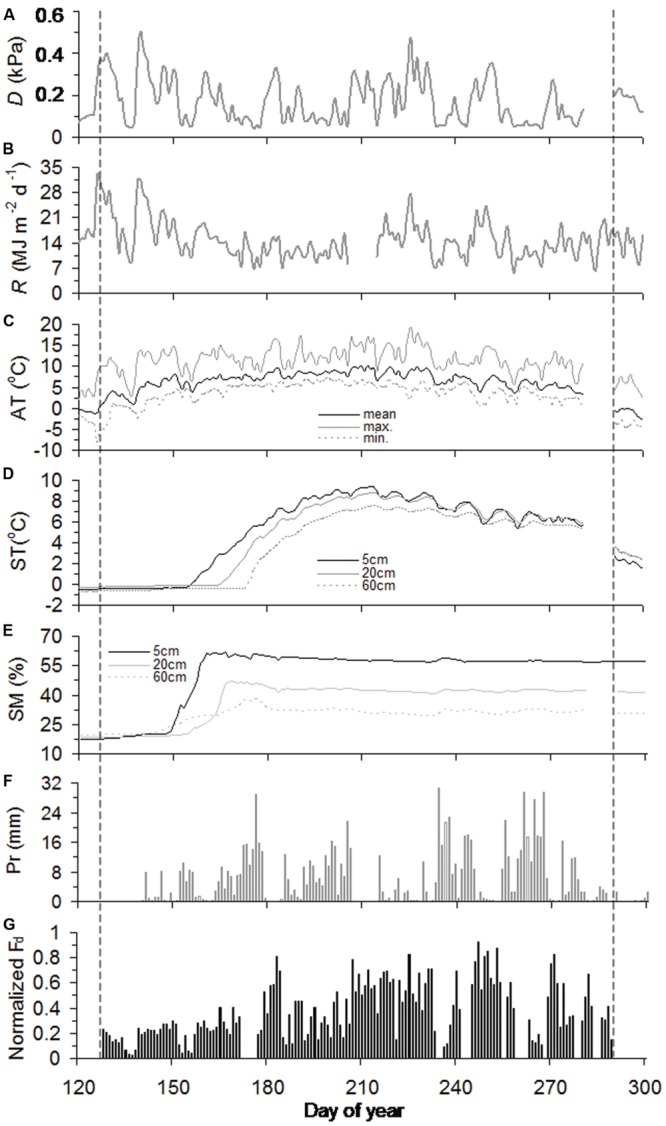
**Seasonal course of vapor pressure deficit (*D*) **(A)**, solar radiation (*R*) **(B)**, air temperature (AT) **(C)**, soil temperature (ST) **(D)**, soil moisture (SM) **(E)**, precipitation (Pr) **(F)**, and normalized daily sum sap flow density (normalized *F*_d_) **(G)** for Smith fir trees during the growing season of 2012.** The vertical black dashed lines highlight the timings of onset and ending of sap flow.

**FIGURE 2:**
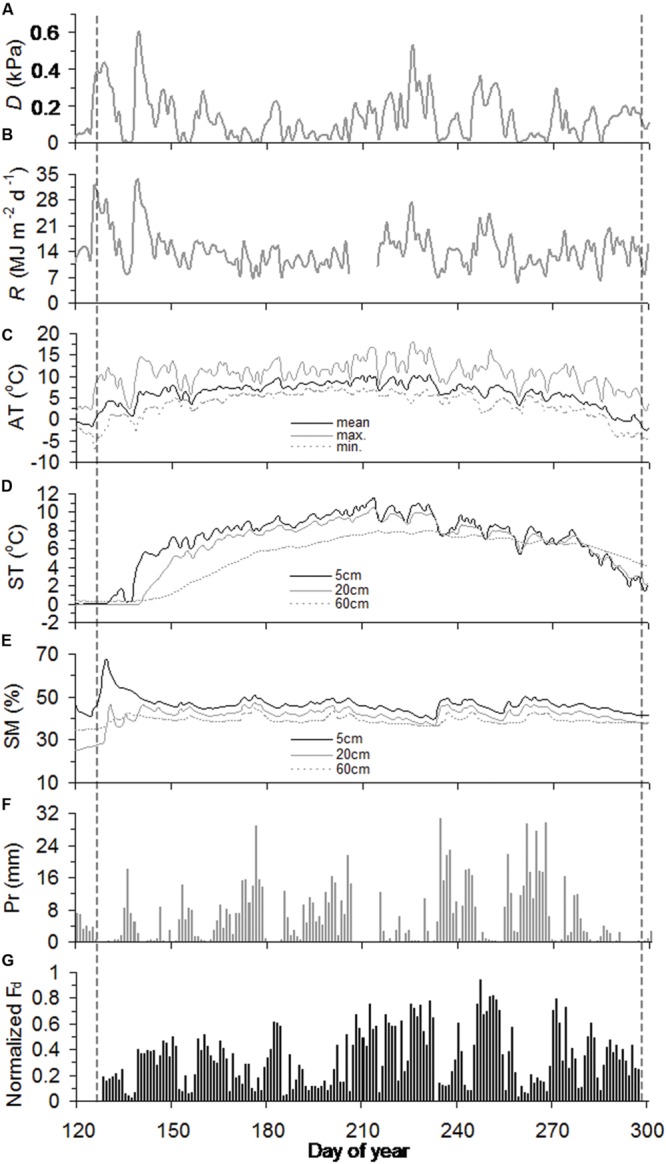
**Seasonal course of vapor pressure deficit (*D*) **(A)**, solar radiation (*R*) **(B)**, air temperature (AT) **(C)**, soil temperature (ST) **(D)**, soil moisture (SM) **(E)**, precipitation (Pr) **(F)**, and normalized daily sum sap flow density (normalized *F*_d_) **(G)** for juniper trees during the growing season of 2012.** The vertical black dashed lines highlight the timings of onset and ending of sap flow.

Across slopes, air temperature and precipitation were generally similar throughout the growing season, while soil warming was delayed into mid-May and early June at the juniper and Smith fir treelines, respectively (**Figures 1C–F** and **2C–F**). Consequently, soil moistures in 20 cm depth sharply increased and stayed unchanged at 40% after thawing at both treelines (**Figures 1E** and **2E**). In contrast, *D* and *R* for both treelines were high from early May to mid-June and then subsequently decreased until late July due to the frequent rainfall (**Figures 1A,B** and **2A,B**).

### Seasonal Pattern in Sap Flow Across Tree Species

In 2012, sap flows of Smith fir and juniper trees simultaneously started in day of year (DOY) 128 and peaked in DOY 247, but asynchronously ended in DOY 289 and DOY 297, respectively (**Figures 1G** and **2G**). The timing of sap flow onset and ending for Smith fir and juniper trees both corresponded to a threshold mean air-temperature of 0°C, while soil temperature still remained around 0°C and soil moisture in 5 cm depth was typically >20% at the beginning of sap flow (**Figures 1** and **2**).

At the seasonal scale, across tree species, there was a considerable difference in normalized *F*_d_ magnitudes between pre- and post-summer solstice. On average, normalized *F*_d_ of Smith fir and juniper trees in pre-summer solstice was significantly lower by 56% (*P* < 0.001) and 23% (*P* < 0.01), respectively, compared to post-summer solstice levels (**Figures 1G** and **2G**).

### Response of Diurnal Sap Flow to *D* over Growing-season Months

**FIGURE 3:**
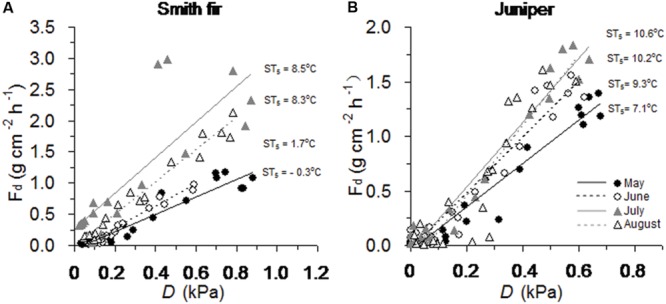
**Relationships between hourly sap flow density (*F*_d_) and vapor pressure deficit (*D*) on bright days over growing-season months across **(A)** Smith fir and **(B)** juniper trees.** Note that mean soil temperature at 5 cm (ST_5_) range from -0.3 to 8.5°C and from 7.1 to 10.6°C for Smith fir and juniper trees, respectively.

Overall, across tree species, the slopes of the relationship between diurnal sap flow density and *D* differed significantly among the four bright days with contrasting soil temperature conditions from May to August (*P* < 0.001, **Table 2**). Particularly, across tree species, the slopes were generally significantly higher in July and August with higher soil temperatures as compared to May and June with lower soil temperatures (*P* < 0.05, **Table 2**; **Figure 3**).

**Table 2:** Differences in slopes of single linear regressions of hourly sap flow density vs. vapor pressure deficit (*D*) across Smith fir and juniper trees over the growing-season months were tested using analysis of covariance (ANCOVA).

Slope	May	June	July	August	*F*-value	*P*-value
Smith fir	0.14^c^	0.18^b^	0.28^a^	0.26^a^	10.92	<0.001
Juniper	0.20^c^	0.25^b^	0.29^ab^	0.32^a^	10.86	<0.001

*F-values and *P*-values of ANCOVA were shown. Pair-wise comparisons were made when the results of ANCOVA were significant. Different letters within a row indicate significant differences in slopes of hourly sap flow density vs. *D* among different growing-season months at *P* < 0.05.*

### Correlations of Sap Flow in Pre- and Post-summer Solstice to Meteorological Factors

**FIGURE 4:**
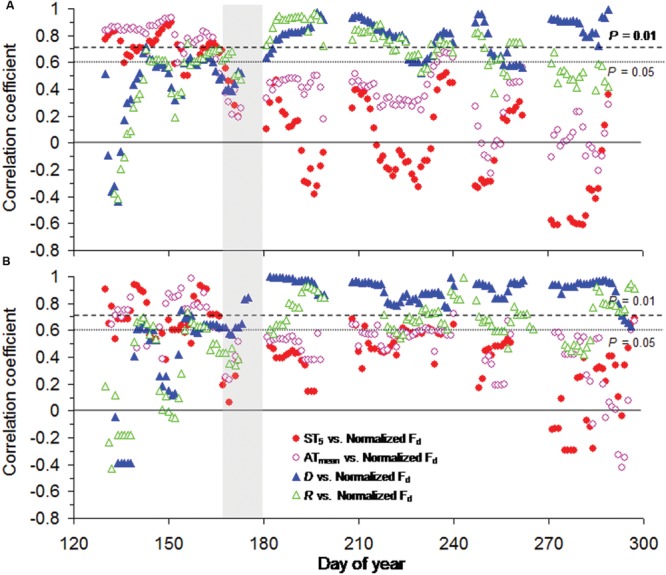
**Moving correlation analysis (10 days window) between normalized daily sum sap flow density (normalized *F*_d_) and meteorological factors for **(A)** Smith fir and **(B)** juniper trees.** The vertical gray areas indicate the period around summer solstice.

Across tree species, moving correlation analysis indicated a strong shift in meteorological limiting factors on tree transpiration around summer solstice. Generally, normalized *F*_d_ for both tree species showed significant relationships with ST_5_ (*P* < 0.05) and AT_mean_ (*P* < 0.05) and an instable response to *D* and *R* before summer solstice (**Figure 4**), though soil temperature at 5 cm depth slightly changed in May and early June, especially at Smith fir treeline (<0.5°C; from **Figure 1D**). In contrast, normalized *F*_d_ for both tree species were continuously correlated with *D* (*P* < 0.01) and *R* (*P* < 0.05) after summer solstice (**Figure 4**).

**FIGURE 5:**
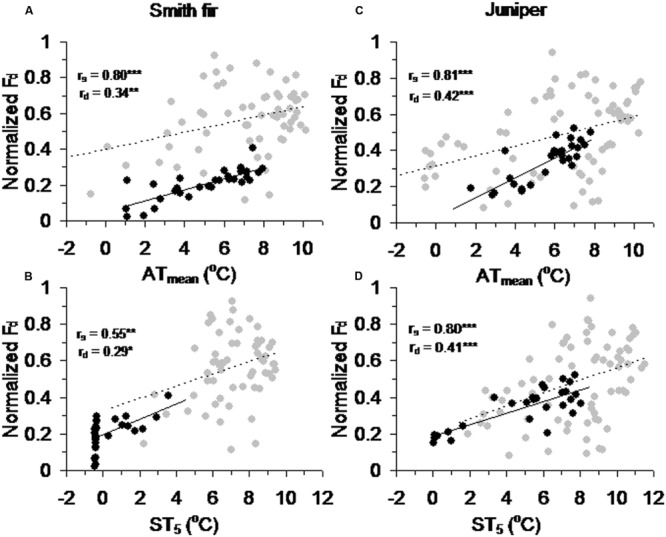
**Correlations between normalized daily sum sap flow density (normalized *F*_d_) across pre- and post-summer solstice growing seasons of Smith fir trees and meteorological factors of **(A)** mean air temperature (AT_mean_) and **(B)** mean soil temperature at 5 cm (ST_5_), and of juniper trees and meteorological factors of **(C)** AT_mean_ and **(D)** ST_5_.** The solid trend line (*r*_s_) is for normalized *F*_d_ in pre-summer solstice (black circle), and the dashed trend line (*r*_d_) is for normalized *F*_d_ in post-summer solstice (gray circle). ^∗^*P* < 0.05, ^∗∗^*P* < 0.01, ^∗∗∗^*P* < 0.001.

**FIGURE 6:**
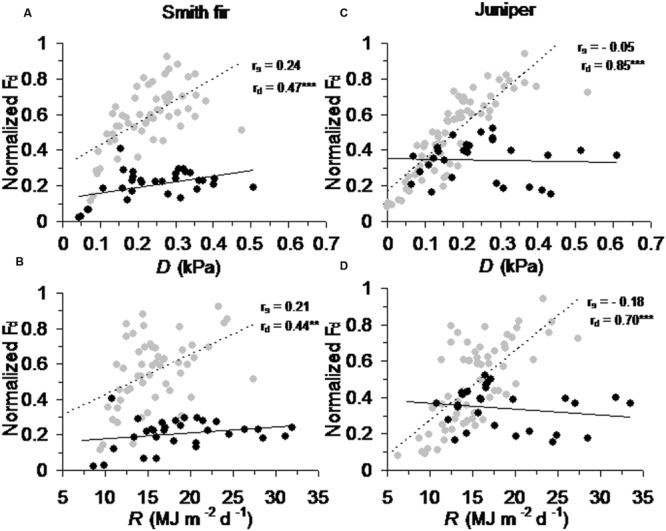
**Correlations between normalized daily sum sap flow density (normalized *F*_d_) across pre- and post-summer solstice growing seasons of Smith fir trees and meteorological factors of **(A)** vapor pressure deficit (*D*) and **(B)** solar radiation (*R*), and of juniper trees and meteorological factors of **(C)***D* and **(D)***R*.** The solid trend line (*r*_s_) is for normalized *F*_d_ in pre-summer solstice (black circle), and the dashed trend line (*r*_d_) is for normalized *F*_d_ in post-summer solstice (gray circle). ^∗^*P* < 0.05; ^∗∗^*P* < 0.01; ^∗∗∗^*P* < 0.001.

Across tree species, normalized *F*_d_ in pre-summer solstice showed a robust positive correlation with AT_mean_ (*P* < 0.001) and ST_5_ (*P* < 0.01) as compared to that in post-summer solstice (**Figure 5**). In contrast, normalized *F*_d_ in post-summer solstice showed a robust positive correlation with *D* (*P* < 0.001) and *R* (*P* < 0.01), while normalized *F*_d_ in pre-summer solstice varied little with *D* and *R* (**Figure 6**). As expected, the partial correlation analysis further indicated that AT_mean_ and ST_5_ mainly determined the variations of normalized *F*_d_ in pre-summer solstice across tree species, while normalized *F*_d_ in post-summer solstice were mainly limited by *D* and *R* (**Table 3**).

**Table 3:** Partial correlation coefficients of multiple linear regressions of normalized daily sum sap flow density (normalized *F*_d_) vs. temperature [mean soil temperature at 5 cm (ST_5_) and mean air temperature (AT_mean_)] and atmospheric evaporative demand [vapor pressure deficit (*D*) and solar radiation (*R*)] variables across tree species and growing seasons.

Independent variables	Smith fir		Juniper	
	Pre-summer solstice	Post-summer solstice	Pre-summer solstice	Post-summer solstice
AT_mean_ and *D*
AT_mean_	0.79^∗∗∗^	-0.34^∗^	0.83^∗∗∗^	0.31^∗∗^
*D*	0.39	0.67^∗∗∗^	0.35	0.84^∗∗∗^
AT_mean_ and *R*
AT_mean_	0.82^∗∗∗^	0.38^∗∗^	0.82^∗∗∗^	0.39^∗∗^
*R*	0.40^∗^	0.50^∗∗∗^	0.27	0.69^∗∗∗^
ST_5_ and *D*
ST_5_	0.73^∗∗∗^	-0.14	0.89^∗∗∗^	0.33^∗∗^
*D*	0.68^∗∗∗^	0.63^∗∗∗^	0.65^∗∗^	0.84^∗∗∗^
ST_5_ and *R*
ST_5_	0.70^∗∗∗^	0.31^∗^	0.84^∗∗∗^	0.32^∗^
*R*	0.56^∗∗^	0.48^∗∗∗^	0.46^∗^	0.67^∗∗∗^

*^∗^*P* < 0.05, ^∗∗^*P* < 0.01, ^∗∗∗^*P* < 0.001.*

### 3.5 Seasonal Changes in Ψ_pd_ of Smith Fir Trees

**FIGURE 7:**
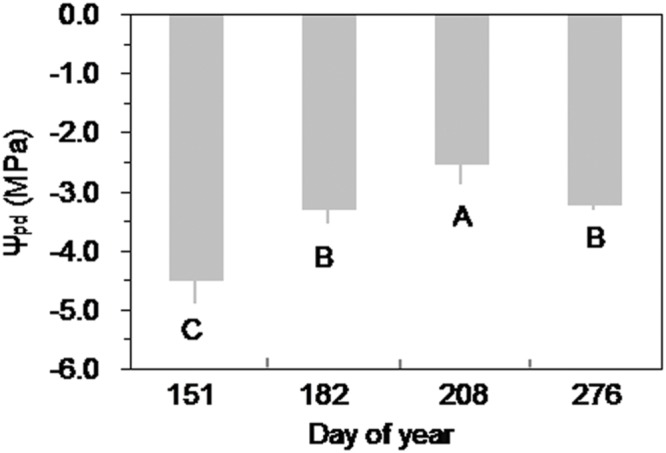
**Seasonal variation of predawn leaf water potential (Ψ_pd_) of Smith fir trees at the treeline sites.** Different letters indicate significant differences in Ψ_pd_ among observed dates at *P* < 0.05. Error bars indicate ±SD of mean.

**FIGURE 8:**
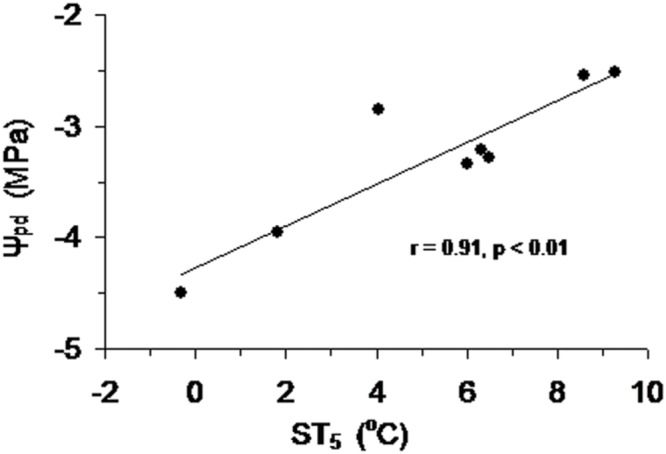
**Predawn leaf water potential (Ψ_pd_) of Smith fir trees at the sub-alpine and treeline sites as a function of mean soil temperature at 5 cm (ST_5_) during the growing season**.

Over the growing season, Ψ_pd_ of Smith fir trees was lowest in late May (*P* < 0.05), indicating that trees faced severe water stress under low soil temperature. In general, Ψ_pd_ showed an increasing trend with increasing soil temperature (**Figure 7**), which was also found at the sub-alpine Smith fir sites (data not shown). Accordingly, Ψ_pd_ was positively correlated with soil temperature in 5 cm depth in pooled data of treeline and sub-alpine sites (**Figure 8**).

## Discussion

### Air Temperature Triggers Sap Flow Onset in Spring

In cold-limited forests, the onset of sap flow has an important influence on the recovery of photosynthesis in spring, and thus annual carbon balance (Bergh and Linder, 1999; Sevanto et al., 2006; Ensminger et al., 2008; Wu et al., 2013). In this study, sap flow of Smith fir and juniper trees both started in early May (**Figures 1G** and **2G**). This is generally in agreement with previous observations at alpine treeline and boreal forests, in which the timing ranged from late April to mid-May (Mayr et al., 2003; Mellander et al., 2004; Wieser et al., 2014, 2015).

Soil thawing has been considered a trigger for transpiration recovery in spring (Mayr et al., 2003; Wieser and Tausz, 2007; Wu et al., 2013). In this study, however, there was no correspondence between sap flow onset and soil thawing at Smith fir treelines, where soil temperature continuously remained around 0°C until early June. Alternatively, our data indicated that mean air-temperature above 0°C was a prerequisite for sap flow onset in spring for both tree species, which also seemed to regulate sap flow completion (**Figures 2C–F** and **2**). This is consistent with a previous report in a boreal forest where photosynthesis and stem diameter variation (reflecting sap flow) were triggered by a rise in air temperature in spring (Sevanto et al., 2006). Physiologically, air temperature above 0°C promotes biochemical reactions in the photosynthetic apparatus and ultimately leads to subsequent transpiration, but frozen soil only reduces the rate of transpiration by restricting water uptake (Ensminger et al., 2008). On the other hand, soil moisture was typically >20% before sap flow onset (**Figures 1E** and **2E**), indicating that soil water availability was not limited by low soil temperature. In fact, it has been demonstrated that in boreal forests, water uptake in roots and the stem based on dendrometer measurements occurred when the soil was partially frozen (Sevanto et al., 2006; Turcotte et al., 2009). Thus, our data suggest that air temperature directly triggers sap flow onset in cold-limited forests. Since sap flow onset and cessation in cold-limited trees were both regulated by a threshold mean air-temperature of 0°C, the delayed sap flow cessation of juniper trees was attributed to differences in air temperature between slopes (**Figures 1C** and **2C**).

### Low Soil Temperature Limits Transpiration in the Early Growing Season

At high latitudes and altitudes, woody plants evolved to maximize photosynthesis and stem growth around summer solstice in order to cope with the cold environments and a short growing season (Rossi et al., 2006; Bauerle et al., 2012), which was also found in Smith fir and juniper trees at Tibetan treelines (Li et al., 2013; XS Liu and TX Luo, unpublished data). Since cold-limited trees are fully active before summer solstice, the concurrent low temperatures in air and/or soil could exert great influence on tree physiological processes. Recently, our data indicated that air temperature in pre-summer solstice mainly determined the intra- and inter-annual variations in stem increments across Smith fir and juniper trees at Tibetan treelines (XS Liu and TX Luo, unpublished data). However, direct evidence for low soil temperature limitation on early-season transpiration at the treeline is still lacking. In this study, averaged transpiration levels in pre-summer solstice were significantly lower than that in post-summer solstice, especially for Smith fir trees. Our data revealed that a strong shift in the main limiting factors that determined variation in transpiration occurred around the summer solstice (**Figure 4**). Under low temperature conditions in pre-summer solstice, transpiration was mainly limited by ST_5_ and AT_mean_ though atmospheric evaporative demand was relatively higher, whereas transpiration was controlled by *D* and *R* when temperature had risen in post-summer solstice (**Figures 5** and **6**; **Table 3**). These findings were further confirmed on a daily scale, in which the hourly sap flow was significantly elevated in mid-to-late summer with warm soil compared to spring–early summer with cold soil at a given *D* (**Figure 3**; **Table 2**). These results are generally in line with previous field experimental data and modeling studies in boreal forests, where low soil temperature severely reduced transpiration in late spring and early summer (Bergh and Linder, 1999; Mellander et al., 2004, 2006; Rainer et al., 2011; Wu et al., 2012).

Cold soil imposes inhibitions on tree transpiration involving increasing water viscosity and decreasing root membrane permeability and aquaporin function, as well as reducing root hydraulic conductance (Kaufmann, 1975; Goldstein et al., 1985; Wan et al., 2001; Mellander et al., 2004). Also, there is evidence that soil temperature <6°C strongly hampers root growth at high altitudes (Alvarez-Uria and Körner, 2007), which may reduce the absorbing surface area of roots. Furthermore, limited water supply due to cold soil will lead to water stress in trees during the early growing season, which had been observed in the Ψ_pd_ of Smith fir tree needles (**Figure 7**). Low needle water potential can promote the release of abscisic acid in shoots and further reduce stomata conductance (Goldstein et al., 1985; Mellander et al., 2004). Conversely, the inhibiting effects of low soil temperature on transpiration will be removed when temperatures increase above a certain threshold. Although such a threshold varies by more than 10°C because of the differences in natural variability and experimental design among tree species (Day et al., 1990, 1991; Schwarz et al., 1997), it has been demonstrated that transpiration is no longer affected by soil temperature when it rises above ca. 8°C (Mellander et al., 2004). For both treelines, soil temperature was almost >8°C after summer solstice, which made trees closely couple with the atmosphere, resulting in a higher Ψ_pd_ of Smith fir trees in post-summer solstice than in pre-summer solstice (**Figure 7**). As a consequence, the effects of atmospheric evaporative demand on the variation of transpiration overwhelm that of temperatures in post-summer solstice. Such mechanisms well explain why there was significantly lower transpiration in pre-summer solstice than in post-summer solstice across tree species. Given the general occurrence of photoperiod induced seasonality in stem growth and related metabolisms, we speculated that trees at both treelines may also use water storage in stems to meet the demand of transpiration in the early growing season (Sevanto et al., 2006).

In alpine and boreal forests, some previous soil warming and cooling studies with manipulating snow cover depth or burying heating cables and soil roofing have consistently reported that early-season transpiration and leaf water potential were significantly increased in heated plots than in unheated control and cooled plots (Bergh and Linder, 1999; Mellander et al., 2004; Wieser et al., 2015). It is well known that low soil temperature limits water uptake through increasing water viscosity and decreasing root permeability, which ultimately determines the water movement along the soil–plant–atmosphere continuum in cold environments (Kaufmann, 1975; Goldstein et al., 1985; Wan et al., 2001; Mellander et al., 2004; Wieser et al., 2015). Also, the functioning of aquaporins is highly sensitive to temperature, with reduced aquaporin activity further inhibiting root hydraulic conductivity under low soil temperature (Wan et al., 2001). Consequently, tree transpiration is restricted because there are positive relationships between leaf-level transpiration and stomatal conductance and root hydraulic conductance (Yang et al., 2011). In general, the field experimental data are in agreement with our findings, suggesting that transpiration of cold-limited forests mainly responds to soil temperature variations in the early growing season.

In southeast Tibet, the unique treeline landscape in which Smith fir and juniper trees dominate opposite slopes of the valley still awaits detail physiological explanations. In this study, the largest difference in sap flow response to environmental factors between tree species occurred in the early growing season, in which sap flow of juniper trees showed a higher sensitivity to *D* and *R* (**Figure 3**; **Table 2**) and was comparable to the level in post-summer solstice compared to Smith fir trees (**Figure 2G** vs. **Figure 1G**). Such contrasting responses were caused by a large difference in snow depth and leaf area index between slopes, leading to an early spring soil warming and consequent higher soil temperature on the south-facing slope (Liu and Luo, 2011). This is in accordance with a previous observation at boreal forests, where delayed soil warming heavily restricted tree transpiration during the early growing season (Mellander et al., 2004). More importantly, the high soil temperature on the south-facing slope accompanied massive diurnal temperature oscillations as compared to the low and stable soil temperature on the north-facing slope (Liu and Luo, 2011). It has been demonstrated that rapid temperature oscillation (i.e., freeze–thaw) under cold environments can induce xylem embolism and cavitation in conifers, which further exerts severe constraints on tree transpiration (Wieser and Tausz, 2007). There is evidence that *Juniperus* spp. are more resistant to cavitation than *Abies* spp. in both roots and stems (Linton et al., 1998; Larcher, 2003). Thus, Smith fir trees may be excluded from the south-facing slope because the pronounced diurnal soil temperature oscillation during the early growing season is fatal to root water uptake and tree transpiration.

## Conclusion

In conclusion, our data supported the hypothesis that in cold-limited forests, the main limiting factors for tree transpiration switch from low soil temperature before summer solstice to atmospheric evaporative demand after summer solstice, which generally results in low transpiration in the early growing season. The findings suggest that tree transpiration mainly responds to soil temperature variations in the early growing season. Globally, the widespread snow cover reduction at high altitudes and latitudes under a warmer winter will lead to delayed soil warming in spring and early summer. Thus, our finding is important for understanding the hydrological response of cold-limited forests to climatic warming. In the future, physiological measurements are needed to further elucidate the underlying mechanism.

## Author Contributions

TL and XL designed the research. XL, JY, WS, and LZ conducted the experiments. XL and TL analyzed and interpreted the data. XL, YN, and TL wrote and revised the manuscript. All authors discussed and approved the final version.

## Conflict of Interest Statement

The authors declare that the research was conducted in the absence of any commercial or financial relationships that could be construed as a potential conflict of interest.
